# Serial survey shows community intervention may contribute to increase in knowledge of Tuberculosis in 30 districts of India

**DOI:** 10.1186/s12889-016-3807-1

**Published:** 2016-11-11

**Authors:** Badri Thapa, Banuru Muralidhara Prasad, Sarabjit S. Chadha, Jamie Tonsing

**Affiliations:** International Union Against Tuberculosis and Lung Disease, C-6, Qutub Institutional Area, New Delhi, 110016 India

**Keywords:** Tuberculosis, Knowledge, Attitude and practice (KAP), India, Community level

## Abstract

**Background:**

Correct knowledge about Tuberculosis (TB) is essential for appropriate healthcare seeking behaviour and to accessing diagnosis and treatment services timely. There are several factors influencing knowledge about TB. The present study was conducted to assess the change in community knowledge of Tuberculosis (TB) and its association with respondent’s socio-demographic characteristics in two serial knowledge-attitude-practice surveys.

**Methods:**

Community level interventions including community meetings with youth groups, village health committees and self-help groups and through mass media activities were undertaken to create awareness and knowledge about TB and service availability. Increase in knowledge on TB and its association with respondent’s socio-demographic characteristics was assessed by two serial KAP surveys in 2010–2011 (baseline) and 2012–2013 (midline) in 30 districts of India. Correct knowledge of TB was assessed by using lead questions and scores were assigned. The composite score was dichotomized into two groups (score 0–6, poor TB knowledge and score 7–13, good TB knowledge).

**Results:**

In baseline and midline survey, 4562 and 4808 individuals were interviewed. The correct knowledge about TB; cough ≥2 weeks, transmission through air, 6–8 months treatment duration, and free treatment increased by 7 % (*p*-value <0.05), 11 % (*p*-value <0.05), 2 % (*p*-value <0.05), and 8 % (*p*-value <0.05) in midline compared to baseline, respectively. The knowledge on sputum smear test for diagnosis of TB was 66 % in both surveys while knowledge on availability of free treatment and that TB is curable disease decreased by 5 % and 2 % in midline (*p*-0.001), compared to baseline, respectively. The mean score for correct knowledge about TB increased from 60 % in baseline to 71 % in midline which is a 11 % increase (*p*-value <0.001). The misconception regarding on transmission of TB by- sharing of food and clothes and handshake persisted in midline. Respondents residing in northern (OR, 2.2, 95 % CI, 1.7–2.6) and western districts (OR, 3.4, 95 % CI, 2.7–4.1) of India and age groups- 25–34 years (OR, 1.3; 95 % CI, 1.1–1.6) and 45–44 years (OR, 1.4; 95 % CI, 1.1–1.7)- were independently associated with good TB knowledge.

**Conclusions:**

The knowledge about TB has increased over a period of 2 years and this may be attributable to the community intervention in 30 districts of India. The study offers valuable lesson for designing TB related awareness programmes in India and in other high burden countries.

## Background

Globally, India is one of the high-burden Tuberculosis (TB) countries contributing to 24 % of estimated new cases and 20 % of TB related deaths in 2013 [1]. The notification rate of all forms of TB (new and relapse) and bacteriologically confirmed cases were 99 and 50 respectively per 100,000 population in 2013 and the notification rate of all TB cases was 113 per 100,000 population. Despite efforts to increase TB case detection, it is estimated that nearly a million cases are being missed in India every year [2]. The missing million could be those who remained un-diagnosed, and not treated for TB or not notified to the programme [3].

In India, private health care facilities are the first and preferred point of contact for 57 % of urban and 48 % of rural population [4]. Almost 50 % of cases detected in private sector are not reported to the programme which is also one of the contributing factor to missing million cases [5]. The lack of awareness and knowledge about symptoms, accurate diagnosis, and treatment of TB hinders the access to free TB services [6]. Deep-rooted stigma and misconceptions to TB also contribute to delay in health care seeking behaviour and prevented people from accessing services [7]. Efforts made by National TB Program (NTP) to establish community level patient centric directly-observed-treatment (DOT) providers was shadowed by stigma and misconceptions towards TB. In addition programme had limited success in engaging local service providers, and community members, to educate people on TB and its free services [8].

Community engagement was on priority and The Global Fund supported TB project -‘Axshya’ (means free of TB in Indian language) (IDA-910-G17-T) was implemented since 2010 to engage civil society to create awareness about TB and the availability of services under NTP [9, 10]. The project activities are primarily aimed at creating awareness and knowledge about TB (symptoms, diagnosis, treatment and availability of free services) among marginalised and vulnerable communities with limited access to TB services in both rural and urban areas. During the first year of project implementation, 2010–11 a baseline survey was conducted to assess the knowledge, attitude and practices (KAP) among key community groups about TB. In the third year of the project 2012–13, a midline survey was conducted to assess the change in KAP and provide evidence-based guidance to identify gaps in implementation and improvise the strategies [9, 10]. The present study analysed the secondary data pertaining to general population from the two serial surveys to assess the knowledge of TB and its association with respondent’s socio-demographic characteristics.

## Methods

### Study settings

The community level intervention activities of project ‘Axshya’ are implemented in 374 districts across 25 states of India [11]. Project districts were selected based on a composite indicators; those districts having higher number of marginalized and vulnerable population, low TB case detection and limited access to TB services. The interventions include activities to create awareness and knowledge about TB and availability of its free services (symptoms, place of free diagnosis and treatment) through community meetings with village health committees, and various community groups including, youth groups, self-help groups, and village head groups. Information about TB was also disseminated through mid-media activities including; community radio, wall painting and street plays. One of the key activities implemented from April 2013 onwards was house-to-house visits by trained community volunteers to inform household members about TB, and to identify presumptive TB patients with cough ≥ 2 weeks and link them with TB services for diagnosis and treatment. From, April 2013 till March 2014, the project has reached five million households and has contributed directly to diagnosis of more than 14,000 smear positive TB patients [12].

### Survey design, sample size, sampling technique and study participants

In 2010–11 and 2012–13, baseline and midline cross-sectional KAP surveys about TB were conducted among general population (excluding TB patients), opinion leaders, non-governmental organizations, health care service providers and TB patients in 30 of the 374 Project Axshya districts [9, 11]. The survey districts were selected through a three staged stratified cluster sampling technique. First, 374 districts were stratified into four zones (north, south, east and west). Of these, 30 representative districts were selected based on population proportion to size (PPS) sampling method. Second, primary sampling units (PSUs)-10 rural and urban villages- were selected in each district using PPS. Third, in each PSU, household listing was carried out which involved assigning numbers to each residential structure, recording address and location of these structures, listing the numbers of individuals in the households, and identification of the head of the household. Then 15 individual respondents (age >18 years, men: women ratio of 1:1) were selected based on the systematic sampling method. To have a statistical power of 80 % to ascertain 2 % or more change at the midline in comparison to baseline, a sample size of 4500 respondents from general population was required. Therefore, sample size with additional 10 % non-response rate was used. The sample size in both surveys was calculated using the same methods [9, 11].

### Data collection, entry and analysis

The study was implemented by The Union, South-East Asia Regional Office, New Delhi, India with assistance from field investigators of the social research organization GfK MODE. The primary sampling units (PSUs) were visited by the trained field investigators during the survey period. The survey included a semi-quantitative questionnaire developed in eight local languages to collect the data and was piloted before being administered to the general population. This semi-structured questionnaire included demographic data on age, sex, settlement (urban/rural), monthly household income (in Indian rupees), literacy status (an illiterate was considered as a person who cannot read and write in any language), and zones. The data on heard of TB, source and preferred source of TB information, knowledge and misconceptions on TB symptoms; mode of transmission, diagnosis, and duration of treatment, ‘completely curable’ and directly observed treatment short course (DOTS) were collected. Data collected were entered into Epi-data (version 2.2.1) and exported into Statistical Package for the Social Sciences (SPSS) version 16 for further analysis. Categorical variables were summarized using proportions and compared using Chi-square test, comparison of mean were done by Student’s t-test and Analysis of variance (ANOVA) and *p*-value of less than 0.05 was considered statistically significant.

For assessing the predictors of good knowledge, a univariate and multivariate logistic regression analysis was performed. The correct knowledge of TB among the study participants was assessed using 13 questions on ‘cough ≥ 2 weeks’, ‘coughing blood’, ‘chest pain’, ‘fever’, ‘weight loss’ and, ‘night sweats’ and ‘loss of appetite’ as symptoms of TB, ‘air’ as a mode of transmission of TB, ‘sputum smear test’ as a diagnostic tool, TB is ‘completely curable, duration of treatment of ‘6–8 months or more’, knows as DOTS and ‘DOTS is free’. Each correct answer was scored “1” and incorrect answer (including don’t know) was scored “0”. The cut off score for correct knowledge was taken at more than 50 % i.e., more than 6 out of 13. The composite score was dichotomized into two groups (score 0–6, poor TB knowledge and score 7–13, good TB knowledge).

## Results

### Respondents and heard of TB

Baseline and midline surveys interviewed 4562 and 4804 individuals, respectively (Table 1). Sex and geographical distribution of the respondents were not statistically significant different between the surveys (*p* > 0.05). There were subtle significant differences in age groups, settlements, education and income level between two surveys (*p* < 0.001) but overall the demographic data were quite similar in both surveys. Of those interviewed, 3822 (84 %) in baseline and 4211(88 %) in midline had heard of TB, a 4 % increase in comparison to baseline survey (*p* < 0.001) (Table 2).

**Table 1:** Socio-demographic characteristics of survey respondents in baseline (2010–2011) and midline (2012–2013) survey in 30 districts in India

Characteristics	Baseline survey		Midline survey		Total		
	Respondents		Respondents		Respondents		
	*n*	%	*n*	%	*n*	%	*p*-value
Total	4562		4804		9366		
Sex							
Women	2242	49	2396	50	4638	50	0.29
Men	2320	51	2408	50	4728	50	
Age (years)							
18–24	692	15	686	17	1378	14	<0.001
25–34	1266	28	1272	31	2538	26	
35–44	1427	31	1238	30	2665	26	
45–54	957	21	865	21	1822	18	
> 55	220	5	743	18	963	15	
Settlement							
Rural	3388	74	3360	70	6748	72	<0.001
Urban	1174	26	1440	30	2614	28	
Education^a^							
Illiterate	1394	31	1110	23	2504	27	<0.001
Literate	3168	69	3668	77	6836	73	
Income							
< 4000	2875	63	2165	45	5040	54	<0.001
> 4001	1580	35	2422	50	4002	43	
Don't know	107	2	217	5	324	3	
Zones							
North	1067	23	1123	23	2190	23	0.98
East	1234	27	1279	27	2513	27	
West	1202	26	1280	27	2482	27	
South	1059	23	1122	23	2181	23	

^a^Education information not available for 26 respondents in midline

**Table 2:** Knowledge of Tuberculosis among respondents in baseline (2010–2011) and midline (2012–2013) surveys in 30 districts in India

Key knowledge’s on TB	Baseline (*n* = 3822)		Midline (*n* = 4211)		Total		***p-value
	*n*	%	*n*	%	*n*	%	
Symptoms*							
Cough of ≥2 weeks	2829	74	3421	81	6250	78	<0.001
Chest pain	1147	30	1138	27	2285	28	0.112
Coughing blood	1721	45	2311	55	4032	50	<0.001
Fever	1262	33	1514	36	2776	35	0.090
Night sweat	77	2	322	8	399	5	0.060
Weight loss	650	17	1075	26	1725	21	<0.001
Loss of appetite	345	9	679	16	1024	13	0.002
Don’t know	421	11	282	7	703	9	0.070
Mode of transmission*							
Air	2293	60	2973	71	5266	66	<0.001
Sharing of food	1184	31	1568	37	2752	34	<0.001
Sharing bed clothes	535	14	875	21	1410	18	0.005
Hand shake	688	18	394	9	1082	13	<0.001
Don’t know	764	20	580	14	1344	17	0.004
Diagnosis*							
Sputum	2523	66	2764	66	5287	66	1.000
Chest X-ray	2332	61	2281	54	4613	57	<0.001
Other (blood, skin, urine)	382	10	59	1	441	5	0.020
Don’t know	573	15	582	13	1155	14	0.100
Curability							
Yes completely	3364	88	3505	83	6869	85	<0.001
Yes partially	305	8	450	11	755	9	0.170
No	38	1	51	1	89	1	1.000
Don’t know	115	3	205	5	320	4	0.390
Duration of treatment							
4wks or less	115	3	97	2	212	3	0.640
1–5mths	612	16	358	9	970	12	0.002
6–8mths	1759	46	2028	48	3787	47	0.220
> 8mths	306	8	777	18	1083	13	<0.001
Don’t know	1030	27	951	23	1981	25	0.124
Knows DOTS	1059	28	1094	26	2153	27	0.043
**Knows DOTS is free	847	80	957	87	1804	22	<0.001
****Heard of TB	3822	84	4211	88	8033	100	<0.001

*Multiple response, **Among those who knew DOTs, ***Chi-square test for comparison of proportions,****Respondents for baseline and midline are 4652 and 4804 respectively. Project is raising awareness on drug sensitive as well as drug resistant tuberculosis and the treatment duration are 6-8 months and >8 months are considered correct

### Correct knowledge and misconception on TB

The knowledge on common symptoms of TB (except chest pain) increased in midline in comparison to the baseline (Table 2). The correct knowledge on the most common symptom of TB- cough ≥ 2 weeks increased from 74 % in baseline to 81 % in midline, a 7 % increase (*p*-value <0.05). Similar significant increase in knowledge were noted in midline survey for, other common symptoms, coughing of blood (45 % vs 55 %), weight loss (17 % vs 21 %) and loss of appetite (9 % vs 16 %) in comparison to baseline (*p*-value <0.05).

The correct knowledge about mode of transmission of TB through air increased from 60 to 71 % in the midline, a 11 % increase (*p*-value <0.001). The misconception that TB could be transmitted through sharing of food and clothes showed a marginal increase in midline (37 % and 21 %) compared to the baseline (31 % and 14 %), respectively (*p*-value <0.05). However, the misconception on transmission through handshake reduced from 18 % in baseline to 12 % in midline (*p*-value <0.001). The knowledge that diagnosis of TB is done by sputum smear examination showed no change in both surveys (66 %, *p* = 1.00). The misconceptions on the diagnostic tests (chest X-ray and others blood and tuberculin test) significantly reduced in midline in comparison to the baseline (*p*- < 0.001).

The response to TB is “completely curable”, decreased to 5 % in midline. The correct knowledge about treatment duration of TB (≥6 months) increased from 54 % in baseline to 66 % in midline (*p*-value <0.001). The knowledge on DOTS decreased by 2 % in midline in comparison to baseline (*p*-value <0.05). Among those who were aware of DOTS, 88 % knew it was available free of cost in midline, compared to 80 % in the baseline (*p*-value <0.05). In all knowledge areas, individuals who said "don’t know" declined in the midline.

The mean score for correct knowledge was 5.1 ± 2.3 in baseline which increased to 5.7 ± 2.2 in midline (*p*-value <0.05) with 12 % increase. The mean score changed slightly for all characteristics in midline in comparison to baseline except for respondents residing in urban areas (Table 3).

**Table 3:** Mean score of TB Knowledge by selected respondent's sociodemographic characteristics in baseline (2010–2011) and midline (2012–2013) surveys in 30 districts of India

Characteristics		Mean TB knowledge		
		Baseline survey*		Midline survey*
Sex				
Female	1806	5	2068	5.5
Male	2016	5.36	2143	5.8
Age (years)				
18–24	609	5.32	629	5.6
25–34	1057	5.08	1134	5.7
35–44	1209	5.09	1068	5.6
45–54	787	5.21	748	5.2
> 55	160	5.34	632	5.6
Settlement				
Rural	2759	4.85	2915	5.5
Urban	1063	6.12	1296	6.0
Education				
Illiterate	989	4.32	889	5.4
literate	2833	5.53	3296	5.8
Income				
< 4000	2291	4.89	528	5.4
> 4001	1531	5.64	3683	5.8
Zones				
North	1020	3.5	990	5.6
East	1050	3.6	1182	5.5
West	979	3.9	1130	6.0
South	773	3.6	909	5.1

*All *p*-value within groups (ANOVA) is <0.001. Range in baseline survey; 0–11; Range in midline survey, 0–13

### Source and preferred source of TB related information

The most common sources of TB related information were television (37 %, 1657) and hospital doctors (37 %, 1665) in baseline and interpersonal communication (58 %) in midline (Table 4). The source of TB related information through all means in midline increased in comparison to baseline. Strikingly, information through interpersonal communication (IPC) changed from 27 % in baseline to 58 % in midline with 115 % increase (*p*-value, <0.001). Television was also preferred source of information in the midline which increased from 44 to 61 % (*p*-value <0.05).

**Table 4:** Source and preferred sources of tuberculosis related information among general population in baseline (2010–11) and midline (2012–13) surveys in 30 districts in India

Variable	Baseline (*n* = 4652)		Midline (*n* = 4804)		Total (*n* = 9456)		*p*-value*
	*n*	%	*n*	%	*n*	%	
Source of TB related information							
Television	1675	37	2697	56	4372	47	<0.001
Hospital doctors	1665	37	2055	43	3720	40	<0.001
Newspaper/magazine/hoarding/posters	1416	31	1730	36	3146	34	0.003
IPC	1233	27	2799	58	4032	43	<0.001
Radio	944	11	584	12	1528	16	0.54
Source of preferred TB related information							
Television	2007	44	2942	61	4949	53	<0.001
Hospital doctors	2185	48	2565	53	4750	51	<0.001
News paper/magazine	1457	31	1176	24	2633	28	<0.001
IPC	2315	51	2916	60	5231	56	<0.001

*IPC* Interpersonal Communication, *Chi-square test for comparison of proportion

### Association of TB knowledge with respondents background characteristics

Univariate logistic regression analysis was carried and those respondent's background socio-characteristics with *p*-value <0.05 were included into the multiple logistic regression analysis to determine the association of respondent’s background characteristics on the outcome measures (good knowledge about TB before [baseline survey] and during [midline survey] the project interventions). The age groups-25–34 years (OR, 1.3), and 35–44 years (OR, 1.4), and respondents residing in North (OR, 2.2), East (OR, 2.1) and West (OR, 3.4) were more likely to have correct TB knowledge in midline than baseline survey (*p*-value <0.05) (Table 5). People with higher income group >4000 Indian Rupees per month had better knowledge about tuberculosis (*p*- < 0.001). The correct TB knowledge was not associated with rural (OR, 0.4 vs 0.8) and illiterate (OR 0.4 vs 0.5) groups in both surveys, the odds of having correct knowledge among these groups improved in the midline albeit at low level.

**Table 5:** Univaraite and Multivariate logistic regression analysis of tuberculosis knowledge by selected respondent's sociodemographic characteristics in baseline (2010–2011) and midline (2012–2013) surveys in 30 districts of India

Characteristics	Number (%) with good TB knowledge	Baseline survey		Number (%) with good TB knowledge	Midline survey	
		OR (95 % CI)¥	OR (95 % CI)€		OR (95 % CI)¥	OR (95 % CI)€
Sex						
Female	472 (26)	0.8 (0.7–0.9)*	0.8(0.7–0.9)*	624 (30)	1.3 (1.1–1.5)*	0.7(0.6–0.9)*
Male	632 (31)	1.0	1.0	783 (37)	1.0	1.0
Age (years)						
18–24	203 (33)	1.0	-	629 (32)	1.0	1.0
25–34	336 (32)	0.9(0.7–1.1)	-	419 (37)	1.3(1.0–1.5)*	1.3(1.1–1.6)*
35–44	319 (26)	0.7(0.6–0.9)	-	380 (36)	1.2(0.9–1.4)	1.4(1.1–1.7)*
45–54	205 (26)	0.7(0.5–0.9)	-	237 (32)	0.9(0.7–1.2)	1.1(0.8–1.4)
> 55	41 (26)	0.8(0.5–1.3)	-	171 (27)	0.7(0.6–1.0)	0.9(0.7–1.2)
Settlement						
Rural	618 (22)	0.4 (0.4–0.5)*	0.4(0.4–0.5)*	891 (31)	1.5(1.3–1.7)*	0.8(0.7–0.9)
Urban	486 (46)	1.0	1.0	516 (40)	1.0	1.0
Education						
Illiterate	159 (16)	0.5 (0.4–0.6)*	0.5 (0.3–0.4)*	167 (19)	2.5(2.1–3.0)*	0.5(0.3–0.6)*
literate	945 (33)	1.0	1.0	1225 (37)	1.0	1.0
Income						
< 4000	537 (23)	0.6(0.5–0.8)*	0.7 (0.5–0.8)*	529 (29)	1.3(1.2–1.5)*	0.7(0.6–0.9)*
> 4001	567 (37)	1.0	1.0	878 (36)	1.0	1.0
Zones						
North	191 (19)	1.1(0.8–1.3)	1.1(0.8–1.4)	315 (32)	1.5(1.2–2.8)*	2.2 (1.7–2.6)*
East	373 (36)	2.3 (1.9–3.0)*	2.0(0.9–3.0)*	374 (32)	1.5(1.2–1.8)*	2.1(1.6–2.5)*
West	380 (39)	2.3 (1.8–2.9)*	2.3(2.2–3.2)*	507 (45)	2.6(2.2–3.2)*	3.4(2.7–4.1)*
South	160 (21)	1.0		211 (23)	1	1.0

*OR* odds ratio, Note: *OR, Odds of having good tuberculosis knowledge of respondents relative to the reference group with an OR of 1.0; ¥, Univariate logistic regression analysis; €, Multivariate logistic regression analysis; In baseline, all demographic variables were significant (*p*-value <0.05) except for age in Univariate logistic regression analysis and were included multiple logistic regression model; In midline, all variables were significant in Univariate logistic regression analysis and were included in logistic regression analysis; Income is per month; Income is in Indian Rupees (1US$ = Rs.62)

## Discussion

The survey results show an increase in the knowledge and awareness of TB among the general population. The correct knowledge in areas like, symptoms, transmission, diagnosis, duration of treatment also considerably increased in the midline. The number of presumptive TB patients examined in the 374 project districts increased from 99,07,457 (2010–2011) to 10,257,051 (2012–2013), a 4 % change [13, 14]. This increase in correct knowledge and improved utilization of TB diagnostic services could be attributed to the community level interventions though this requires more evidence to establish association.

Knowledge about chest pain as symptoms, TB is completely curable, and DOTS did not change much which requires focused messages during the ongoing interventions. The misconception on the diagnostic tests “chest X-ray, blood examination and skin test” was reduced but the misconception that TB spreads “through sharing food and clothes” still persists, also needs additional attention. Small scale studies published from India have shown that the awareness of TB (94 %), and knowledge on cough (73 %, 82 %), mode of transmission (65 %, 81 %), sputum test (40 %), and duration of treatment (6.9 %) have varied in different settings and population studied [15–17]. Unlike those studies, these serial surveys have shown that the level of awareness and knowledge on TB has increased over 2 years of community level interventions.

Most common source of TB related information was television and healthcare providers in baseline which changed to Interpersonal Communication (IPC) followed by television in the midline. Television was the preferred source of information for TB in midline which reflects the potential of mass media strategies to educate the community. The television users among the respondents increased from 59 % in baseline to 77 % in the midline which might have indirectly influenced the increase source of information as television but this needs additional investigation. In a similar setting in Bangladesh, mass-media intervention by government and Bangladesh Rural Advancement Committee (known as BRAC) found to have increased awareness at community level [18]. IPC as the commonest source of information in the midline could be due to the project interventions like, community meetings and house-to-house visits by community volunteers and sensitization of community health care providers who are the first point of contact to almost half of the population in the intervention districts [11, 13, 14]. IPC as preferred source of TB related information has been described as an effective strategy and has resulted to increase visits to NTP services [19]. This justifies the importance of strategizing communication channels like IPC among vulnerable and marginalized communities. Multiple logistic regression analysis was performed to determine the association of respondent’s socio-characteristics on the outcome measures (correct knowledge). There were significant difference in age, settlement, education and income of respondents between two survey. In both survey, the respondents were selected taking into consideration the sex (1:1) and geographic region (eastern, northern, western and southern). However the age, settlement, education, and income were not among the selection criteria. This could be the reason for differences. In both surveys, the association of correct knowledge was seen with individuals residing in eastern and western zones. The association was significant for north in the midline survey and also for the older age groups (25–34, 35–44 years).Contradicting to our findings, older age groups (>30 years) were less likely to have high knowledge than young in a community in Ethiopia [20]. The respondents in East had good TB knowledge in baseline survey and the association did not increase in midline. Other projects and ongoing effort of NTP might also have contributed to this increase in knowledge.

The survey showed that women did not have good TB knowledge as compared to men. In midline this was more remarkable. Also coverage in all areas of knowledge among women was less than 50 % in both surveys. This could be partly due to a men dominated society in India where women hesitate to interact with strangers or even community health care workers. Gender inequality in health (including TB) is a major issue in India and gender sensitive communications interventions are to be planned within the project [21]. Illiterates were less likely to have good TB knowledge and need to develop strategies to increase awareness. Similar to the finding in our study, women and illiterates were less likely to have high TB knowledge in a survey in Ethiopia [20]. IPC is a preferred source of TB related information among the participants and this strategy could be effectively used to increase TB knowledge in the women and illiterates.

Despite increase in TB knowledge in the midline, there are numerous bottlenecks in implementation which includes wide and diverse geographical coverage, transportation inequalities, semantic barrier, socio cultural inequalities, standardization of messages, non-uniform communication channels, and varied capacity of NGOs, CBOs and community volunteers, and non-uniform NTP services. There are some limitations in this study: (i) the study represents 374 project districts and the data presented here cannot be generalized for the entire nation, (ii) the participants in the two surveys were different, (iii) the study is powered at the zonal level, and (iv) increase in TB knowledge could also be due to other intervention and projects, NTP efforts and due to increase in education and economic level of the respondents interviewed at midline. Further research is needed to understand cause effect relationship in the project with increase in knowledge and changes in TB services seeking behaviour, presumptive TB patient examination and TB patient diagnosed in NTP disaggregated by age, gender and socio-economic factors.

## Conclusion

The knowledge of TB among general population in midline survey considerably increased than baseline however misconception on the mode of transmission and diagnosis still prevailed which needs to be strengthened through interpersonal communication. Correct knowledge was associated with, males, older age group, high income, literacy and individuals residing in north, east and west. Community level interventions for TB prevention and care in India, have possibly contributed to the increased TB knowledge which could be replicated in other high burden countries.

## Abbreviations

CBO: Community Based Organization
CV: Community volunteer
DOTS: Directly observed treatment short course
IPC: Interpersonal communication
KAP: Knowledge Attitude Practice
NGO: Non-government organization
NTP: National Tuberculosis Programme
TB: Tuberculosis
